# Accurate inference of isoforms from multiple sample RNA-Seq data

**DOI:** 10.1186/1471-2164-16-S2-S15

**Published:** 2015-01-21

**Authors:** Masruba Tasnim, Shining Ma, Ei-Wen Yang, Tao Jiang, Wei Li

**Affiliations:** 1 Department of Computer Science and Engineering, University of California, Riverside, Riverside, CA, 92507, USA; 2 MOE Key Lab of Bioinformatics and Bioinformatics Division, TNLIST / Department of Automation, Tsinghua University, Beijing, 100084, China; 3 MOE Key Lab of Bioinformatics and Bioinformatics Division, TNLIST / Department of Computer Science and Technology, Tsinghua University, Beijing, 100084, China; 4 Department of Biostatistics and Computational Biology, Dana-Farber Cancer Institute and Harvard School of Public Health, Boston, MA, 02215, USA

## Abstract

**Background:**

RNA-Seq based transcriptome assembly has become a fundamental technique for studying expressed mRNAs (*i.e*., transcripts or isoforms) in a cell using high-throughput sequencing technologies, and is serving as a basis to analyze the structural and quantitative differences of expressed isoforms between samples. However, the current transcriptome assembly algorithms are not specifically designed to handle large amounts of errors that are inherent in real RNA-Seq datasets, especially those involving multiple samples, making downstream differential analysis applications difficult. On the other hand, multiple sample RNA-Seq datasets may provide more information than single sample datasets that can be utilized to improve the performance of transcriptome assembly and abundance estimation, but such information remains overlooked by the existing assembly tools.

**Results:**

We formulate a computational framework of transcriptome assembly that is capable of handling noisy RNA-Seq reads and multiple sample RNA-Seq datasets efficiently. We show that finding an optimal solution under this framework is an NP-hard problem. Instead, we develop an efficient heuristic algorithm, called Iterative Shortest Path (ISP), based on linear programming (LP) and integer linear programming (ILP). Our preliminary experimental results on both simulated and real datasets and comparison with the existing assembly tools demonstrate that (i) the ISP algorithm is able to assemble transcriptomes with a greatly increased precision while keeping the same level of sensitivity, especially when many samples are involved, and (ii) its assembly results help improve downstream differential analysis. The source code of ISP is freely available at http://alumni.cs.ucr.edu/~liw/isp.html.

## Introduction

Transcriptomic research has taken advantage of recent high-throughput sequencing methods, leading to a new experimental protocol, RNA-Seq [1]. A major application of RNA-Seq is transcriptome assembly and isoform (or transcript) abundance estimation, where full-length mRNA transcripts and their expression levels are inferred from RNA-Seq data. Transcriptome assemblies can help analyze both structural and quantitative differences of expressed isoforms between samples. Such (differential) analysis could, for example, lead to the detection of oncogenes that are associated with cancers [2] and splicing variants that are responsible for diseases [3].

If a reference genome is available, transcriptome assembly usually begins by mapping RNA-Seq reads to the reference genome. After that, different algorithms can be used to infer transcripts from mapped reads, including Cufflinks [4,5], IsoInfer [6], IsoLasso [7], SLIDE [8], CLIIQ [9], MITIE [10], *etc*. This *ab initio *approach is different from the *de novo *approach where reference genome is not used (such as AbySS [11], Trinity [12], *etc*.), and is able to take advantage of information provided by the reference genome. As a result, *ab initio *assemblers are able to recover transcripts with a better accuracy and yet demand less computational resource [13]. However, their results critically depend on the quality of the reference genome and mapping software, and they are not specifically designed to handle errors [13], which come from various sources including unwanted RNA fragments during the library preparation, mapping errors (due to sequencing errors and/or repeats), or "dark matters" from inter-genetic and intron regions [14].

In many RNA-Seq based studies, multiple sample RNA-Seq datasets are available. It is now common for an RNA-Seq project to sequence the whole transcriptomes of samples obtained from multiple replicates, tissues, populations, *etc*. For example, the ENCODE project [15] aims at creating functional element profiles of more than 100 human cell lines, and more than 200 RNA-Seq datasets from various tissues and experimental protocols are available for public use [16]. Other large research projects (including TCGA [17], modENCODE [18], *etc*.) are also producing many multiple sample RNA-Seq data. On one hand, RNA-Seq reads from multiple samples could potentially help assemble transcripts better than reads from only one sample, since the samples can be correlated. On the other hand, transcriptome assembly for multiple samples and subsequent differential analysis are more challenging because (i) multiple sample RNA-Seq data typically contains more noise and (ii) differential analysis is very sensitive to assembly and abundance estimation errors. Therefore, to analyze the structural and quantitative differences of isoforms from multiple samples, a highly accurate transcriptome assembly and abundance estimation tool is necessary.

A straightforward way to assemble transcriptomes for multiple samples is to "merge" all transcripts that are assembled from individual samples as a "universal" set of isoforms, which is then used for downstream applications including abundance estimation and differential analysis. An example of this approach is the "Cuffmerge" program in the Cufflinks software package [5]. However, as more samples are sequenced, errors from individual assemblies are likely to accumulate, which could seriously affect the isoform abundance estimation and result in unreliable (or even misleading) differential analysis results.

In this paper, we present a new framework for *ab initio *transcriptome assembly that is able to handle noisy RNA-Seq reads and multiple sample RNA-Seq datasets effectively. Instead of assembling transcripts separately for each sample and merging them together, our framework reconstructs transcripts directly from multiple samples. In fact, it takes advantage of the extra information contained in paired-end reads and in multiple sample RNA-Seq datasets (*e.g*., correlation among the samples). We show that finding an optimal solution under this framework is NP-hard, and develop a heuristic algorithm, called ISP (for *Iterative Shortest Path*), to reconstruct isoforms efficiently under the framework. For a given gene, ISP solves either a linear programming (LP) or an integer linear programming (ILP) problem iteratively on a weighted graph derived from the input multiple sample RNA-Seq dataset. Our preliminary experimental results on both simulated and real datasets demonstrate that (i) ISP is able to assemble transcriptomes with high precision and sensitivity, especially when many samples are involved, and (ii) the assembly results of ISP help improve downstream differential analysis.

## Methods

### Multiple Sample Connectivity Graph (MSCG)

A set of RNA-Seq reads from *F *different samples are first mapped independently to the reference genome using a splice junction detection tool such as Tophat [19], SpliceMap [20], *etc*. The mapped reads are then clustered into genes, and the exon-intron boundary information for each may be derived from either its junction reads or existing annotations such as NCBI RefSeq [21] or UCSC known isoforms [22]. Based on this information, the sequence of a gene can be split into different *expressed segments *(or simply *segments*) [6], where a segment is a continuous region in the reference genome uninterrupted by any splicing events (or exon-intron boundaries).

**Figure 1:**
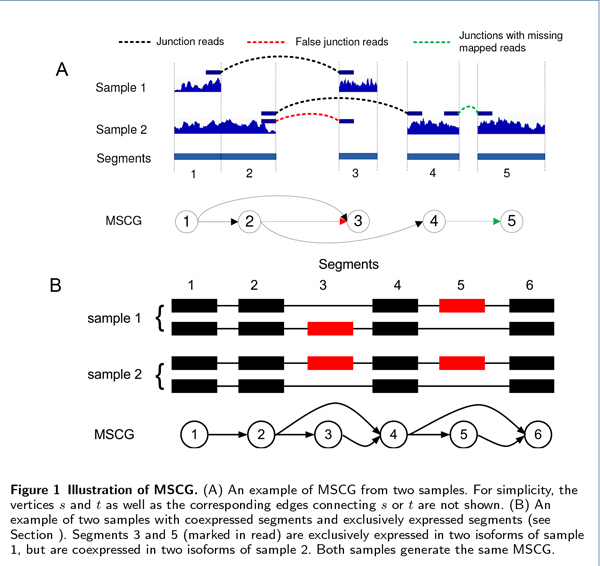
**Illustration of MSCG**. (A) An example of MSCG from two samples. For simplicity, the vertices *s *and *t *as well as the corresponding edges connecting *s *or *t *are not shown. (B) An example of two samples with coexpressed segments and exclusively expressed segments (see Section). Segments 3 and 5 (marked in read) are exclusively expressed in two isoforms of sample 1, but are coexpressed in two isoforms of sample 2. Both samples generate the same MSCG.

Several transcriptome assemblers [7,9,23] use the *Connectivity Graph (CG) *to represent the splicing connections between segments or bases on single sample RNA-Seq data. Similarly, for multiple sample RNA-Seq data, we construct a *multiple sample connectivity graph (MSCG) G *= (*V, E*) based on *F *sets of mapped RNA-Seq reads as follows. *V *= {*s, t*} ∪ {*v_i_*|1 ≤ *i *≤ *M*} where *v*_1_,... *v_M _*represents the *M *segments contained in a gene. (*v_i_, v_j_*) ∈ *E *if there is at least one read from the *F *samples joining both segments *i *and *j*. Also, (*s, v_i_*) ∈ *E*, (*v_i_, t*) ∈ *E*, 1 ≤ *i *≤ *M *(see Figure 1(A)).

For simplicity, *s *is assigned number 0, *t *is assigned number *M *+1, and the vertices *v*_1_,...*v_M _*are all assigned numbers 1 through *M*. Thus, a vertex in *V *or an edge in *E *can be represented as a tuple (*i, j*) with 0 ≤ *i, j *≤ *M *+ 1. For example, (0, *i*) = (*s, v_i_*) and (*i, M *+ 1) = (*v_i_, t*) for 1 ≤ *i *≤ *M*. Similarly, (*i, j*) ∈ *E *if (*v_i_, v_j_*) ∈ *E *for *i *≠ *j*, and (*i, j*) ∈ *V *if *i *= *j *(1 ≤ *i, j *≤ *M*).

### Isoform discovery on MSCG

In MSCG, every path *P *from *s *to *t *represents a possible isoform of the gene. We further assign weights to each vertex (or edge) in MSCG to represent the probability that the corresponding segment (or junction) is included in an isoform; the higher the weights, the lower probability that they are included. As a result, a shortest path (minimum weight path) represents the most possible isoform of the gene. Furthermore, we make use of paired-end read information (if any) and expression correlation between segments among the samples that could help the reconstruction of isoforms.

Our approaches to assigning weights and utilizing information from paired-end reads and segment expression correlations are described in detail below.

#### Weight assignment

A weight *w_i,j _*is assigned for each edge (*i, j*) ∈ *E *(if *i *≠ *j*) and each vertex *v_i _*∈ *V *(if *i *= *j*) to reflect the likelihood that the corresponding segment (or junction) is "problematic": a higher weight is assigned if the segment (or junction) is more likely the product of some incorrectly mapped reads. Notice that *w_i,j _*may be either positive (considered as "cost") or negative (considered as "reward"). For simplicity, the weight of a path *P *is represented as), $\sum_{\left(i,j\right)\in P}w_{i,j}$, which is the sum of all weights of the vertices and edges included on *P*.

Assume that the same number of reads are generated from *F *samples. For every vertex *v_i _*∈ *V*\{*s, t*}, we assign *w_i,i _*= − log(*d_i _*+ 1), where *d_i _*is the average read density of segment *i *in the *F *samples:

(1) $$d_{i}=\overset{F}{\underset{k=1}{\sum }}C_{i}^{k}/\left(l_{i}-L+1\right)$$

Here, $C_{i}^{k}$ is the number of reads mapped to segment *i *from the *k*th RNA-Seq sample, *l_i _*is the length of the segment *i *and *L *is the read length. Since we will look for a shortest path, paths going through segments with high densities are preferred.

Because noisy junctions may result in incorrect assembly results, a higher positive cost is assigned for junction edges that are more likely to be problematic. For every edge (*i, j*) ∈ *E*, where 1 ≤ *i, j *≤ *M *and *i *≠ *j*, we set

(2) $$w_{i,j}=-\text{log}P\left(i,j\right)=-\text{log}\left(\frac{d_{i,j}}{\sum_{h}d_{i,h}}\frac{d_{i,j}}{\sum_{h}d_{h,j}}\right)$$

where 1 ≤ *h *≤ *M*, *P *(*i, j*) represents the probability that the junction between segments *i *and *j *is included in an isoform, and *d_i,j _*is the average read density of edge (*v_i_, v_j_*) ∈ *G*:

(3) $$d_{i,j}=\overset{F}{\underset{k=1}{\sum }}C_{i,j}^{k}/\left(L-1\right)$$

where $C_{i,j}^{k}$ is the number of reads mapped to the corresponding junction from the *k*th RNA-Seq sample.

For every edge (0, *i*) ∈ *E*, we "inactivate" it by setting *w*_0,*i *_to infinity if *v_i _*can be reached from another vertex *v_j _*in the MSCG *G*; otherwise, we set *w*_0,*i *_= 0. Similarly for edge (*i, M *+ 1) ∈ *E*, *w*_*i,M *+ 1 _is assigned infinity if there is an edge from *v_i _*to another vertex *v_j _*in the MSCG *G*, or 0 otherwise.

#### Incorporating paired-end read information

Paired-end RNA-Seq reads provide more information than single-end reads in transcriptome assembly, since both ends of a paired-end read come from the same RNA (or cDNA) fragment. To incorporate paired-end read information into our framework, we try to find a path in MSCG to simultaneously minimize the cost of the path and maximize the number of paired-end reads that are *compatible *with the isoform represented by the path. Reads that are compatible with an isoform are the reads that are possibly generated from the isoform. If a read is compatible with an isoform, the splicing patterns implied by the read and the isoform must be identical. More precisely, a single-end read *b *containing *k *segments can be represented as a vector *b *= (*b*_1_*, b*_2_,⋯, *b_k _*), where 1 ≤ *b*_1 _< ⋯ <*b_k _*≤ *M *are the segments included in *b*. An isoform *I *(or a path *P *) that *b *is compatible with must include all the segments *b*_1_,⋯, *b_k _*, and must not include any other segment between *b*_1 _and *b_k_*. A paired-end read *p *= (*b, b′*) is compatible with *I *if and only if both *b *and *b′ *are compatible with *I*.

For each paired-end read *p *= (*b, b′*), where *b *= (*b*_1_,⋯ *b_k _*) and $b^{\prime }=\left(b_{1}^{\prime },\cdots b_{k}^{\prime }\right)$, we define the set of "inclusion segments" *IS_p _*and "exclusion segments" *ES_p _*as follows:

(4) $$IS_{p}=b\cup b^{\prime }$$

(5) $$ES_{p}=\left\{i:b_{1}<i<b_{k}\;\;\text{or}\;\;b_{1}^{\prime }<i<b_{k}^{\prime },i\notin IS_{p}\right\}$$

Intuitively, *IS_p _*(and *ES_p_*) represents the set of segments that an isoform *I *must (and must not) include, based on the information of *p*. For example, if *p *= ((*b*_1_*, b*_3_), (*b*_5_*, b*_7_)), then *IS_p _*= {*b*_1_*, b*_3_*, b*_5_*, b*_7_} (as they are included in *p*), and *ES_p _*= {*b*_2_*, b*_6_} (as they are spliced out in *p*). For each paired-end read *p*, we define a binary variable *q_p~P _*∈ {0, 1} to indicate whether *p *is compatible with a path *P *implied in the solution. Given a set of paired-end reads *R*, maximizing the number of compatible reads with *P *is equivalent to maximizing), $\sum_{p\in R}q_{p\sim P}$. For each gene, paired-end reads that are mapped to only one segment (*i.e*., |*IS_p_*| = 1) are excluded from *R*, since these reads do not provide any useful information in the assembly.

#### Resolving ambiguities using Jensen-Shannon metric

In a complicated gene model, an MSCG may give rise to several sets of isoforms due to the existence of segments that introduce ambiguities (named as "uncertain" segments). For example, the MSCG in Figure 1(B) has two edges at each end of segment 4 due to the two uncertain segments, segments 3 and 5. Different combinations of these pairs of edges would lead to two possible sets of isoforms. Paired-end reads can be used to resolve such ambiguity (as in [23]), but it only works if there are paired-end reads mapped to uncertain segments. In [5], isoforms are decomposed such that the expression levels of the segments in one isoform are similar, but this strategy does not consider positional biases [24] and is applied only to a single sample.

In this work, we use *Jensen-Shannon metric *(or JS metric) to resolve the ambiguity of uncertain segments. JS metric measures the similarity of the expression patterns between samples and was used to analyze differential alternative splicing events [5]. It is defined as the square root of the *Jensen-Shannon divergence *[25]:

(6) $$JS\left(i,j\right)={\left(H\left(\frac{p_{i}+p_{j}}{2}\right)-\frac{H\left(p_{i}\right)+H\left(p_{j}\right)}{2}\right)}^{1/2}$$

where *H*(*x*) stands for the entropy of the probability distribution *x *and *p_i _*is the distribution of segment *i *among the samples. The latter is calculated based on the read density of segment *i *(defined in Equation (1)) over all *F *samples.

If the JS metric of the expression levels of two uncertain segments is low (which means both segments are positively correlated), then both segments are likely to be included on the same isoform (termed "coexpressed segments", see Figure 1(B) for an example). Otherwise if the JS metric is high, they are likely to appear in different isoforms (termed "exclusively expressed segments"). To determine whether two uncertain segments are coexpressed or exclusively expressed, we randomly permute the expression of each segment in a gene 1000 times, and calculate the "background" JS metric distribution *P_bg_*. For a given false-discovery rate (FDR) *β*% (controlled by the user; the default is 5%), segments *i *and *j *are considered coexpressed (or exclusively expressed) if *J S*(*i, j*) is located in the lowest (or highest) *β*% of *P_bg_*, respectively. For coexpressed segments *i *and *j*, we add some "pseudo" paired-end reads *p_c _*spanning segments *i *and *j *(*i.e*., *IS_pc _*= {*i, j*}) to the read set *R*. These reads will encourage our algorithm (*i.e*., ISP) to prefer paths that include both segments *i *and *j*. Similarly for exclusively expressed segments *i *and *j*, paired-end reads *p_e _*with *IS_pe _*= {*i*} and *ES_pe _*= {*j*} are added to *R*.

### The objective function and complexity of the problem

Using the notations defined in previous sections, given an MSCG *G *and a single-end and/or paired-end read set *R*, our objective function is to find a path *P *from *s *to *t *in the MSCG to maximize

(7) $$\underset{\left(i,j\right)\in P}{\sum }-w_{i,j}+\underset{p\in R^{\prime }}{\sum }\alpha_{p}q_{p\sim P}$$

where the set *R′ *includes all "pseudo" reads and excludes all reads *p *∈ *R *with |*IS_p_*| = 1. Here, *α_p _*> 0 is a user-defined parameter and should be smaller for organisms with simple splicing patterns (like fruit fly or warm) and relatively larger for organisms with more complicated splicing patterns (like human or mouse). For the convenience of presentation, we will refer to this problem as a (constrained) *shortest path *problem on *G*, because when *α *= 0, the problem reduces to finding the minimum weight path (or shortest path) from *s *to *t*.

Unfortunately, it is hard to find a path to maximize Equation (7), since we can show that the corresponding decision problem is NP-complete even when *w_i,j _*= 0 and *α_p _*= 1.

**Theorem**: The following decision problem is NP-complete:

Input: An MSCG *G *= (*V, E*) and a set of mapped paired-end reads *R*; an integer *k*.

Question: Is there a path *P *in *G *such that), $\sum_{p\in R}q_{p\sim P}\geq k$?

**Proof **: The theorem can be proven by a straightforward reduction from the wellknown CLIQUE problem. The reduction is presented in Additional file 1.   ■

### An efficient heuristic algorithm to identify expressed isoforms in multiple samples

#### The ILP and LP approaches for finding an optimal path

In this section, we present two different approaches to find a path on the MSCG *G *maximizing Equation (7). First, a binary variable *f *(*i, j*) ∈ {0, 1} is introduced to indicate whether each vertex (or edge) in *G *is included in a path *P*. The following ILP problem is formulated to find a path maximizing Equation (7):

(8) $$max\;\;\;\;\;\;\;\;\;\;\;\;\;\;\;\;\;\;\;\;\underset{0\leq i,j\leq M+1}{\sum }-w_{i,j}f\left(i,j\right)+\underset{p\in R^{\prime }}{\sum }\alpha_{p}q_{p}$$

(9) $$\text{s}\text{.t}\;\overset{M}{\underset{i=1}{\sum }}f\left(0,i\right)=1,1\leq i\leq M$$

(10) $$\underset{0\leq k\leq M}{\sum }f\left(i,M\right)=f\left(i,i\right)=\underset{0\leq k\leq M}{\sum }f\left(k,i\right),1\leq i\leq M$$

(11) $$q_{p}\leq f\left(i,i\right),i\in IS_{p}$$

(12) $$q_{p}\leq 1-f\left(i,i\right),i\in ES_{p}$$

(13) $$f\left(i,j\right),q_{p}\in \left\{0,1\right\},0\leq i,j\leq M+1$$

Equations (9)-(10) are constraints ensuring that the final solution represents a path (and thus an isoform) from *s *to *t*, while Equation (11)-(12) guarantee that *q_p _*= 1 if and only if the path *P *is compatible with paired-end read *p*. Solving the above ILP problem may be time-consuming since the number of variables may be large for some genes. Instead, we could relax the binary constraints in Equation (13) as follows, which turns the problem into an LP problem:

(14) $$0\leq q_{p},f\left(i,j\right)\leq 1$$

Ideally, the solution to the above LP problem is integral (*i.e*., *q_p_, f*(*i, j*) ∈ {0, 1}), which would represent a path from *s *to *t*. However, in some cases (for about 0.1% of the genes in our simulated and real data experiments), the LP problem may not lead to an integral solution. For these genes, we can solve the corresponding ILP problem instead. We use GNU Linear Programming Kit (GLPK, [26]) to solve both the ILP and LP problems.

#### The Iterative Shortest Path algorithm

A gene may have multiple isoforms expressed in the samples, but only one isoform is extracted by solving the above LP/ILP problem. To recover more expressed isoforms of the gene, we apply the "weight-decay" strategy [27] to modify the weights in the graph *G *and iterate the algorithm several times. In each iteration, the weights are adjusted to encourage the algorithm to look for an isoform different from all previously found isoforms. The details of this ISP algorithm are described in Additional file 1.

## Results

### Simulation results

We simulated RNA-Seq reads and evaluated the performance of different algorithms following the method described in [7,28]. Briefly, we used UCSC known human (and mouse) transcripts [22] to simulate single-end and paired-end reads and evaluate the *sensitivity *and *precision *of different assemblers on noisy RNA-Seq data and multiple samples. Following the definition in [6] and [7], two transcripts are matched if their exon coordinates are identical except the start of the first exon and the end of the last exon. If *K *of *M *predicted transcripts match *K *of *N *known transcripts, then the sensitivity and precision are defined as *K/N *and *K/M *, respectively. We added two different types of noisy reads in the simulation to capture noise in real RNA-Seq data: *noisy junction reads *and *noisy intron reads*. Noisy junction reads are generated by randomly shifting the splicing positions of some normal junction reads by 1 to 3 bases. These reads are added since in reality, splicing regulators may shift the splice site a few bases to the proximal or distal intron boundaries [29,30]. Noisy intron reads are reads coming randomly from the intron regions of a transcript. They are added since it has been observed that a fair amount of reads come from intronic regions in practice, possibly due to intron retention, non-coding RNAs or other unknown mechanisms [14].

We compared the performance of ISP with two existing assembly algorithms for multiple samples, Cufflinks/Cuffmerge [4,5] and MITIE [10]. Cufflinks and Cuffmerge are algorithms incorporated in the Cufflinks software package. For multiple RNA-Seq samples, Cufflinks first constructs a set of isoforms from multiple samples, followed by Cuffmerge merging assembly results from each individual sample. MITIE, on the other hand, constructs isoform structures by solving a mixed integer programming problem defined on multiple samples. CLIIQ [9] is another recent tool for assembling isoforms from multiple sample RNA-Seq data based on integer programming. However, we have had great difficulty in getting CLIIQ to run on our servers (even with the help of the authors of CLIIQ). Hence, we will make a comparison with CLIIQ indirectly and present the comparison results in Additional file 1.

#### The effect of noisy RNA-Seq reads on single sample data

**Figure 2:**
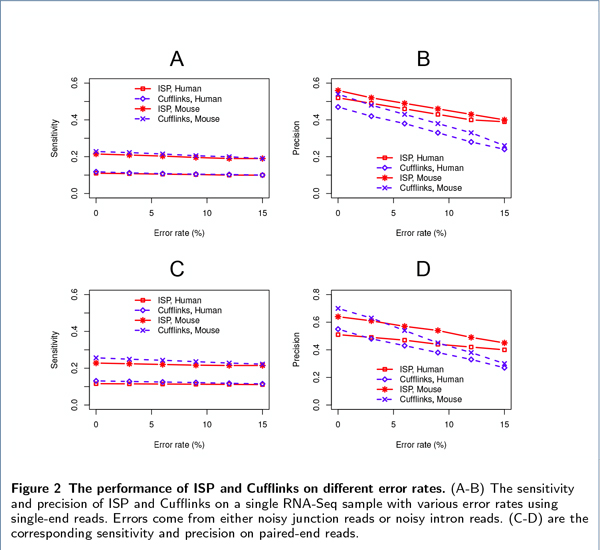
**The performance of ISP and Cufflinks on different error rates**. (A-B) The sensitivity and precision of ISP and Cufflinks on a single RNA-Seq sample with various error rates using single-end reads. Errors come from either noisy junction reads or noisy intron reads. (C-D) are the corresponding sensitivity and precision on paired-end reads.

We added different amounts of noisy reads of both types to a single sample RNA-Seq dataset, and the sensitivity and precision of ISP and Cufflinks are presented in Figure 2. Here, a total of 80 million single-end or paired-end reads are used, and "error rate" shows the percentage of the randomly shifted junction reads and noisy intron reads added to the dataset. When more errors are added, both programs keep the same level of sensitivity (about 10%), but the precision of both programs gradually drops. Compared with Cufflinks, ISP is less affected by the errors, showing that ISP is able to handle read errors better on single sample RNA-Seq data.

It is worth noting that when the simulated RNA-Seq data is error-free, mapping tools may still result in incorrectly mapped reads and thus the input to Cufflinks/ISP could still be noisy. Also, the low sensitivity of both programs is due to the fact that many of the transcripts are assigned very low expression levels (or they are not expressed at all) based on the log-normal model [31]. These transcripts with few (or no) mapped reads decrease the value of sensitivity.

#### Assembly for multiple sample RNA-Seq data

To compare the performance of these algorithms on multiple sample RNA-Seq data, we generated six RNA-Seq datasets with different numbers of samples and evaluate the sensitivity and precision of the programs. For each dataset, the expression level of an isoform is independently assigned and 10% noisy reads are added as errors. To reconstruct all isoforms from multiple samples, a straightforward algorithm is to merge the RNA-Seq reads from all samples together and apply a transcriptome assembly tool (such as Cufflinks) designed for single sample RNA-Seq data. As a comparison to ISP and Cuffmerge, we also tested Cufflinks and ISP on pooled data where RNA-Seq reads from all samples are merged together.

**Figure 3:**
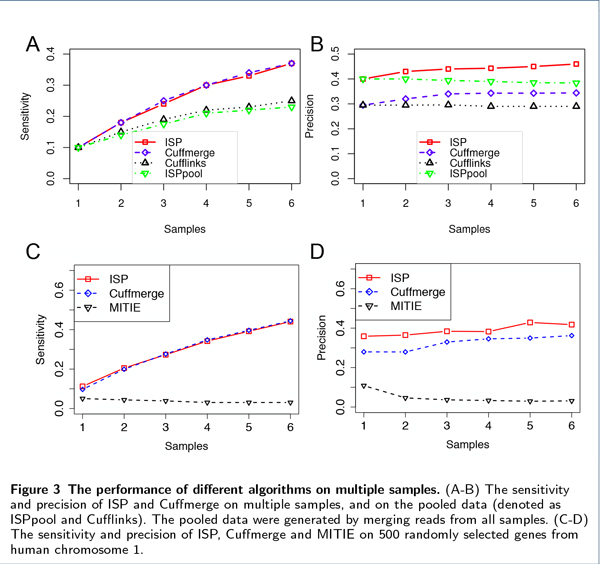
**The performance of different algorithms on multiple samples**. (A-B) The sensitivity and precision of ISP and Cuffmerge on multiple samples, and on the pooled data (denoted as ISPpool and Cufflinks). The pooled data were generated by merging reads from all samples. (C-D) The sensitivity and precision of ISP, Cuffmerge and MITIE on 500 randomly selected genes from human chromosome 1.

Figure 3(A-B) shows both sensitivity and precision of the four programs on different numbers of samples. When only one sample is considered, the sensitivity of all programs is the same. As more samples are added, more transcripts are correctly predicted, and both ISP and Cuffmerge achieve similar improvements of sensitivity on six samples. As for the precision, ISP has a clear advantage, maintaining 40% to 60% higher values than Cuffmerge, and 60% to 80% higher values than Cufflinks. The increasing trend of sensitivity and precision for both ISP and Cuffmerge shows that both programs are able to take advantage of the existence of multiple samples and improve their sensitivity and precision simultaneously. Instead, the precision of Cufflinks and ISP on the pooled data (denoted as Cufflinks and ISPpool in the figure) drops slightly while their sensitivity falls behind ISP and Cuffmerge. This is because as reads from more samples are merged, the detectable splicing patterns become more complicated. Although more isoforms can be discovered (thus improving the sensitivity), many incorrect isoforms are also predicted (thus hurting the precision) because of the increased difficulty in dealing with complex splicing patterns. Therefore, the straightforward approach for dealing with multiple samples is not a good way to treat multiple sample RNA-Seq data.

MITIE [10] is a recently published algorithm that assembles transcripts from multiple samples. Since MITIE uses mixed linear programming to infer isoforms, it requires very long execution time and large memory space to process human RNA-Seq datasets. For practical considerations, we compared MITIE with ISP and Cuffmerge on RNA-Seq samples that were simulated from 500 randomly selected genes on human chromosome 1, including 1206 annotated transcripts (or 2.41 transcripts/gene). Figure 3(C-D) shows the sensitivity and precision of different programs on different numbers of samples. Both ISP and Cuffmerge achieve similar sensitivity and precision in these tests with smaller samples, compared with Figure 3(A-B) using the whole transcriptome for the simulation. MITIE has much lower levels of sensitivity and precision (below 12%), and both values do not increase as more samples are used.

#### Transcriptome assembly and differential analysis

In a typical differential analysis, we are interested in finding and ranking genes (or isoforms) that are differentially expressed between two samples (or two groups of samples). Since isoforms assembled from individual samples may be different, it is necessary to construct a "universal" set of isoforms from all samples, from which the expression level estimation and statistical analysis can be performed. For example, Cufflinks includes a set of programs for differential analysis, and all of them are based on merging isoforms from individual assemblies (using Cuffmerge).

We are interested in the effect of multiple sample transcriptome assembly on differential analysis. We simulated two RNA-Seq datasets and generate a set of isoforms for both samples by running (i) ISP and (ii) Cufflinks followed by Cuffmerge. To avoid using different expression level estimation methods preferred by both methods, we used Cuffdiff 2 [5], the differential expression analysis tool in Cufflinks package, for expression level estimation and differential analysis (including fold change calculation and statistical evaluation) after running ISP and Cuffmerge.

**Figure 4:**
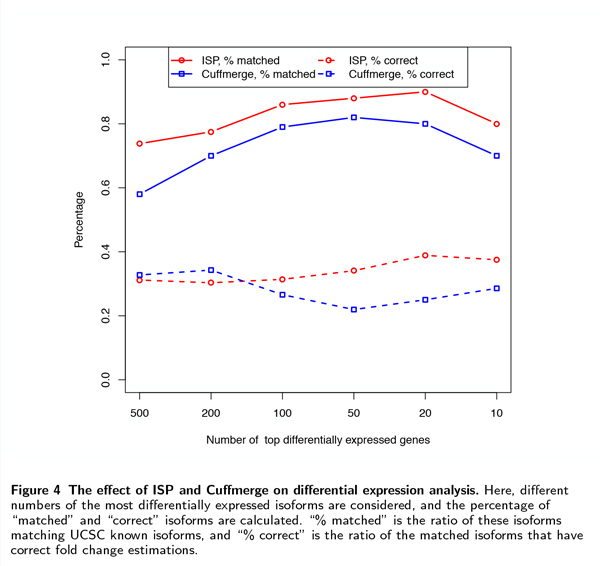
**The effect of ISP and Cuffmerge on differential expression analysis**. Here, different numbers of the most differentially expressed isoforms are considered, and the percentage of "matched" and "correct" isoforms are calculated. "% matched" is the ratio of these isoforms matching UCSC known isoforms, and "% correct" is the ratio of the matched isoforms that have correct fold change estimations.

We selected different numbers of isoforms that show the greatest changes of expression levels. Figure 4 shows the percentage of isoforms that match UCSC human known genes, and the percentage of the matched ones that have correct fold change estimations (defined as estimated fold changes within the [-2,+2] range of their corresponding true fold changes). We showed the trends as we decrease the number of selected isoforms, since we usually prefer finding fewer isoforms with higher expression changes between samples.

ISP is able to find a larger number of matched (*i.e*., true) isoforms than Cuffmerge when more than 10 isoforms are selected. This is consistent with the previous experiments showing a higher precision of ISP than Cuffmerge. Furthermore, ISP outputs more isoforms with correct fold change estimations. With different numbers of top ranked isoforms selected, 74%-90% have their fold changes correctly identified, which is higher than Cuffmerge (58%-82%). Because we use the same algorithm (Cuffdiff 2) for expression level estimation and differential analysis, we suspect that the low precision of Cuffmerge assembly led to the low accuracy in expression level estimation, hence reducing its performance in fold change estimation.

### Real RNA-Seq data results

To compare the performance of the algorithms on real RNA-Seq data, we used the public RNA-Seq datasets of 7 cancer cell lines downloaded from the ENCODE project [32]. These cell lines (GM12878, H1-hESC, K562, HeLa-S3, HepG2, HUVEC, NHEK; NCBI GEO accession code: GSE23316) include normal and cancer cells of different tissues, and are the major cell models extensively used in biological and biomedical research [16].

#### Transcriptome assembly results

It is difficult to measure exactly which isoform is expressed in real RNA-Seq data since the current experimental techniques limit the ability to detect full-length transcripts efficiently. Instead, we treat all UCSC known transcripts as "canonical" isoforms and calculate both sensitivity and precision with respect to these isoforms, the same as in the simulation experiments.

**Figure 5:**
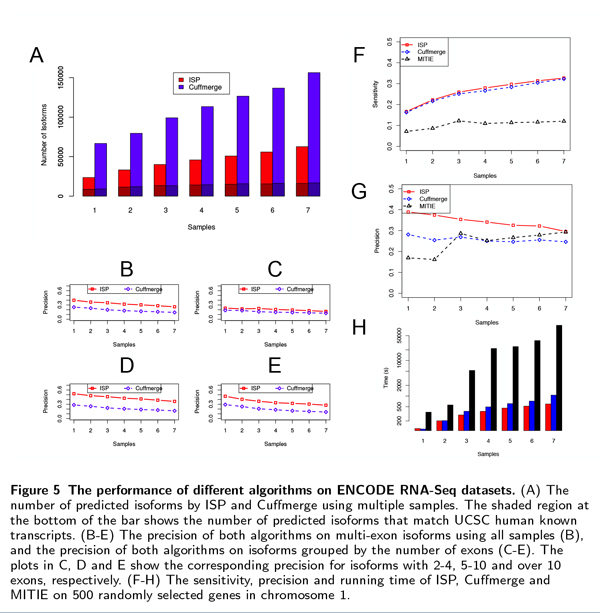
**The performance of different algorithms on ENCODE RNA-Seq datasets**. (A) The number of predicted isoforms by ISP and Cuffmerge using multiple samples. The shaded region at the bottom of the bar shows the number of predicted isoforms that match UCSC human known transcripts. (B-E) The precision of both algorithms on multi-exon isoforms using all samples (B), and the precision of both algorithms on isoforms grouped by the number of exons (C-E). The plots in C, D and E show the corresponding precision for isoforms with 2-4, 5-10 and over 10 exons, respectively. (F-H) The sensitivity, precision and running time of ISP, Cuffmerge and MITIE on 500 randomly selected genes in chromosome 1.

Figure 5(A) shows the numbers of predicted isoforms, together with the numbers of matched UCSC known transcripts by using different numbers of samples. For a single sample, the number of isoforms predicted by Cuffmerge is over 60, 000, which is approximately twice as much as ISP. As more RNA-Seq samples are added, more transcripts are merged by Cuffmerge, and this number reaches 150, 000 (over 100% growth) when all seven samples are included. In contrast, ISP shows a moderate increase, with only 40% more predicted isoforms for seven samples compared to using only one sample. However, the numbers of matched UCSC known transcripts remain roughly the same for both programs, with ISP achieving over 90% of the number attained by Cuffmerge. This illustrates that ISP is able to keep a high precision while sacrificing sensitivity a little when the number of samples increases. An example transcriptome assembly results by ISP and Cuffmerge on some ENCODE data can be found in Section 4 of Additional file 1.

Cuffmerge predictions include a large number of single-exon transcripts that do not match any UCSC known transcripts. To study the effect of multiple samples on the inference of multi-exon isoforms, we exclude these single-exon transcripts and calculate the precision for isoforms grouped by their numbers of exons (see Figure 5(B-E)). ISP shows a higher precision than Cuffmerge on all multi-exon isoforms, and when all seven samples are used, its precision is almost doubled compared to Cuffmerge (see Figure 5(B)) for isoforms containing 5-10 exons. For isoforms with more than 10 exons, the difference between the two algorithms becomes smaller, but ISP still maintains a 70% higher precision than Cuffmerge (Figure 5(D-E)). Isoforms with more exons are difficult to assemble since more errors may occur around splice junctions (see Section 5 of Additional file 1). As a result, the high precision of ISP may be attributed to its ability to handle noise effectively and to use information from multiple samples.

We also compared ISP, Cuffmerge and MITIE on RNA-Seq reads from 500 randomly selected genes from human chromosome 1, similar to the comparisons in the simulation tests. The test was performed on a linux cluster node with a 4-core 2.50GHz CPU and 16G memory. Figure 5(F-H) show the sensitivity, precision and running time of the three algorithms on different numbers of samples. In contrast to the simulation tests, MITIE has a much better performance, achieving similar levels of precision with ISP and Cuffmerge when more than two samples are used. However, the sensitivity of MITIE is still lower than those of ISP and Cuffmerge, and it takes much longer time to run, especially when more samples are used (Figure 5(H)). For example, MITIE takes 163.7X longer than ISP (and 92.4X longer than Cuffmerge) to run when 7 samples are used, making it difficult to use on datasets with a large number of samples, especially when the large transcriptomes such as human or mouse are being studied.

#### Differential analysis

The expression profiles of some ENCODE cell lines have been measured by both RNA-Seq and Affymetrix Human Exon 1.0 ST Array (NCBI GEO accession code: GSE19090). To validate the differential analysis results from RNA-Seq reads, transcripts are assembled from two cell lines (GM12878 and K562) that have corresponding microarray data, and their expression level changes are compared with the microarray measurements. An Affymetrix Human Exon Array uses "probesets" (*i.e*., sets of probes) to measure the expression levels of exons. To calculate the expression levels of transcripts, we only keep probesets whose measured exons correspond to only one RefSeq transcript (called "unique" probesets). For those RefSeq transcripts that include at least one such unique probeset, their expression levels are calculated by averaging the measurements of all unique probesets. As in the simulation experiments, we used Cuffdiff 2 for expression level estimation and differential analysis.

ISP and Cuffmerge are able to identify 4468 and 4627 transcripts that have corresponding expression level estimations in the microarray data, respectively. Comparing the fold change calculations with the microarray data, ISP assembly results reach a higher PCC (Pearson Correlation Coefficient) value than Cuffmerge (0.68 vs 0.58). To further compare the differential analysis results, we select transcripts that show the largest fold changes between samples (similar to the simulation experiments). For the corresponding microarray measurements of these transcripts, we used Student's t-test to check the statistical significance that these transcripts are differentially expressed between both samples. Table 1 shows the PCC values of fold change calculations between RNA-Seq and microarray measurements, and the numbers of top ranked differentially expressed transcripts confirmed by microarray data. The fold change calculations based on the assembly results of ISP are more accurate since they achieve higher PCC values than Cufflinks, and a higher number of predictions are confirmed by microarray measurements. This shows that by using the isoforms inferred by ISP, we are able to obtain a more accurate differential analysis than Cuffmerge.

**Table 1:** The correlation to microarray fold change calculations, and the numbers of differentially expressed isoforms confirmed by microarray measurements among the top ranked isoforms.

Top isoforms		10	50	100	200
PCC	ISP	0.95	0.86	0.87	0.86
	Cuffmerge	0.89	0.82	0.82	0.81

confirmed (*p *< 0.05)	ISP	9	45	94	170
	Cuffmerge	8	43	82	148

confirmed (*p *< 0.001)	ISP	8	35	77	135
	Cuffmerge	7	32	64	108

## Conclusion

With the advance of next generation sequencing technologies, it is now possible to reconstruct full-length transcripts, estimate their expression levels, and compare the structural and quantitative differences between samples. Transcriptome assembly may benefit from the existence of multiple sample RNA-Seq data, but may also be confused by inherent RNA-Seq errors, which in turn affects downstream differential analysis. In this paper, we have designed an algorithm (ISP) to reconstruct transcriptomes for multiple sample RNA-Seq data that is able to handle errors effectively by using an iterative linear programming (or integer linear programming) approach. Both simulated and real experimental results demonstrate that, obtaining a set of accurately assembled transcripts is crucial for downstream differential analysis. A large number of false positives decrease the accuracy of estimating the expression fold changes of isoforms between samples, and ISP is able to achieve a better differential analysis performance by accurately assembling transcripts from multiple samples directly.

## Competing interests

The authors declare that they have no competing interests.

## Supplementary Material

Additional file 1 **Supplementary Materials**. This file includes the description of the ISP algorithm, the NP-completeness proof of the ISP problem, the indirect comparison between ISP and CLIIQ, an example of transcriptome assembly results on ENCODE samples, and the comparison of detecting alternative splicing events between ISP and Cuffmerge. Click here for file

## Declarations

The research was partially supported by National Science Foundation grant DBI-1262107.

This article has been published as part of *BMC Genomics *Volume 16 Supplement 2, 2015: Selected articles from the Thirteenth Asia Pacific Bioinformatics Conference (APBC 2015): Genomics. The full contents of the supplement are available online at http://www.biomedcentral.com/bmcgenomics/supplements/16/S2
