# Supplementary material for: Accurate inference of isoforms from multiple sample RNA-Seq data
Source: BMC Genomics. 2015 Jan 21;16(Suppl 2):S15. doi: 10.1186/1471-2164-16-S2-S15 (PMC4331715; doi:10.1186/1471-2164-16-S2-S15)
Supplement: Additional file 1 — Supplementary Materials. This file includes the description of the ISP algorithm, the NP-completeness proof of the ISP problem, the indirect comparison between ISP and CLIIQ, an example of transcriptome assembly results on ENCODE samples, and the comparison of detecting alternative splicing events between ISP and Cuffmerge. [file 1471-2164-16-S2-S15-S1.pdf]

Supplementary Materials for Accurate Inference of Isoforms from Multiple Sample RNA-Seq Data

Masruba Tasnim[1] and Shining Ma[2,1] and Ei-Wen Yang[1] and Tao Jiang[1,3*] and Wei Li[4,1*]

[1] Department of Computer Science and Engineering, University of California, Riverside, Riverside, CA 92507, USA
[2] MOE Key Lab of Bioinformatics and Bioinformatics Division, TNLIST/Department of Automation, Tsinghua University, Beijing 100084, China
[3] MOE Key Lab of Bioinformatics and Bioinformatics Division, TNLIST/Department of Computer Science and Technology, Tsinghua University, Beijing 100084, China
[4] Department of Biostatistics and Computational Biology, Dana-Farber Cancer Institute, Harvard School of Public Health, Boston, MA 02215, USA
wli@jimmy.harvard.edu jiang@cs.ucr.edu

1 The ISP algorithm

The details of ISP are described in Algorithm 1. The value of W determines how much error can be tolerated in the solution. A larger value of W tends to result in more isoforms (thus recovering more true ones), but isoforms with higher weights are more likely to be erroneous. The values of the parameters W, λ, γ can be determined empirically using simulation. In our experiments, we set $W = 0$, $\lambda = 1.1$ and $\gamma = 1.1$.

Algorithm 1 ISP

Require: a MSCG G, the paired-end read set $R = \{p\}$, $W > 0, \lambda > 1$, $\gamma > 1$.
1: Initialize the weights $w_{i,j}$, $0 \leq i, j \leq M+1$; S = {}.
2: Solve the LP or ILP problem and find a path P from s to t with weight $W_P = \sum_{(i,j)\in P} w_{i,j}$.
3: **if** $W_P > W$ **then**
4: Terminate the algorithm and return S.
5: **end if**
6: Convert the path P to an isoform I, and remove all reads that are compatible with I in R.
7: **if** $I \notin$ S **then**
8: Set S = S $\cup \{I\}$.
9: **end if**
10: **for** $0 \leq i, j \leq M+1$ **do**
11: **if** $w_{i,j} \geq 0$ **then**
12: Set $w_{i,j} = \lambda w_{i,j}$;
13: **else**
14: $w_{i,j} = w_{i,j}/\lambda$.
15: **end if**
16: **end for**
17: **for** each edge or vertex (i, j): **do**
18: **if** the corresponding segment (or junction) is already used in S **then**
19: **if** $w_{i,j} \geq 0$ **then**
20: Set $w_{i,j} = \gamma w_{i,j}$;
21: **else**
22: Set $w_{i,j} = w_{i,j}/\gamma$.
23: **end if**
24: **end if**
25: **end for**
26: Go to Line 2.

2 The complexity of the ISP problem

In this section, we show that finding a path to maximize Equation (7) is NP-hard. Consider the following ISP problem with a special MSCG $G_0 = (V_0, E_0)$ satisfying three conditions:

- $(i, j) \in E_0$ for every $i, j \in V_0 \backslash \{s, t\}$, $i \neq j$;
- $w_{i,j} = 0$;
- $\alpha_p > 0$.

From the construction of G_0, for every non-empty subset of vertices $V_0' \subseteq V_0$ in G_0, there is a path from s to t that connects all vertices (and only vertices) in V_0'. Also, maximizing Equation (7) is equivalent to maximizing $\sum_{p \in R} q_{p \sim P}$ (or the number of compatible paired-end reads with P).

The following theorem holds true:

Theorem 1. *The following decision problem is NP-complete:*
Input: An MSCG $G_0 = (V_0, E_0)$ and a set of mapped paired-end reads R; an integer k.
Question: Is there a path P in G such that $\sum_{p \in R} q_{p \sim P} = k$?

Proof. The theorem can be proven by a reduction from the well-known CLIQUE problem.

First, given an MSCG G_0 and a read set R with m reads, construct a graph $G^H = (V^H, E^H)$ such that:

- $V^H = \{v_1^H, \ldots v_m^H\}$;
- $(v_i^H, v_j^H) \in E^H$ if and only if $IS_i \cap ES_j = \emptyset$ and $IS_j \cap ES_i = \emptyset$.

We show that for each path P, the read set $\{p \in R : p \sim P\}$ correspond to a clique $C = \{v_p^H, p \sim P\}$ in G^H such that

$$\sum_{p \in R} q_{p \sim P} = |C| \tag{1}$$

This is because for any $p, q \in R$ satisfying $p \sim P$ and $q \sim P$, we have $IS_p \cap ES_q = \emptyset$ and $IS_q \cap ES_p = \emptyset$. As a result, $(v_p^H, v_q^H) \in E^H$ and C is a clique.

Next, for an arbitrary graph $G^H = (V^H, E^H)$, we construct a MSCG G_0 and a read set R. We show that for every clique C in G^H, there exists a path P satisfying Equation (1). Let $V^H = \{v_1^H, v_2^H, \ldots v_m^H\}$ and we construct $G_0 = (V_0, E_0)$ as follows:

- $V_0 = \{s, t\} \cup \{v_{i,j}, 1 \leq i, j \leq m, i \neq j\}$;
- $(p, q) \in E_0$ for every $p, q \in V_0$ except (s, t). As usual, s is labelled 0 and t labelled $m(m-1)/2 + 1$.

R is constructed as follows: $R = \{r_1, \ldots r_m\}$ and let the Inclusion and Exclusion Set of read r_i as $IS(r_i)$ and $ES(r_i)$, respectively. For every vertex $v_{i,j} \in V_0 \backslash \{s, t\}$, if $(v_i^H, v_j^H) \in E^H$, then

$$v_{i,j} \in IS(r_i), v_{i,j} \in IS(r_j); \tag{2}$$

otherwise if $(v_i, v_j) \notin E^H$, then

$$v_{i,j} \in IS(r_i), v_{i,j} \in ES(r_j). \tag{3}$$

Suppose $C = \{c_1^H, \ldots c_k^H\}$ is a clique in G^H. Now we show that the corresponding read set $R' = \{r_c, c \in C\}$ and a path P connecting all vertices $IS_P = \{v_{i,j} : v_{i,j} \in IS(r_c), c \in C\}$ (but not other vertices) satisfy Equation (1).

First of all, according to the construction of G_0, there is a path connecting all vertices of P. Next, let $ES_P = \{v_{i,j} : v_{i,j} \in ES(r_c), c \in C\}$, then $IS_P \cap ES_P = \emptyset$. Otherwise if $v_{i,j} \in IS_P \cap ES_P$, then $v_{i,j} \in IS(r_i), v_{i,j} \in ES(r_j)$, or $v_{i,j} \in IS(r_j), v_{i,j} \in ES(r_i)$. In either case, c_i^H and c_j^H are not connected according to the construction of R, which contradicts the fact that C is a clique. As a result, we have $r \sim P$ for all $r \in R'$, and $|R'| = k = |C|$. Therefore, Equation (1) holds. □

3 Comparison with CLIIQ

CLIIQ [1] is a recently developed tool for assembling transcripts directly from multiple samples. To compare the performance of CLIIQ and ISP indirectly (since we were unable to run CLIIQ on our servers, perhaps due to software incompatibility issues), we followed the simulation experiments in the CLIIQ paper and compare the performance of ISP with the results shown in [1]. Simulated RNA-Seq data were generated as described in [1]. More specifically, 770 genes containing 2 to 10 exons were chosen from chromosomes 1-4 in the UCSC human hg19 gene annotation. For the corresponding 2151 isoforms from these 770 genes, 50 bp single-end RNA-Seq reads were generated at the coverage of 30X.

Two different experiments were performed following [1]. In the first experiment, all known isoforms of each gene were expressed and in the second experiment, at most two isoforms of each gene were expressed.

We compared ISP and CLIIQ in terms of precision, recall and F-score which were used as the evaluation metrics in [1]. Notice that the definition of precision (and recall) in the CLIIQ paper is identical to the definition of precision (and sensitivity, respectively) in our paper, and F-score is defined as:

$$F - score = \frac{2 \times precision \times recall}{precision + recall} \tag{4}$$

Table S1. The performance of CLIIQ and ISP in the first experiment, where all known isoforms of each gene were expressed.

	CLIIQ		ISP	
	1 sample	5 samples	1 sample	5 samples
precision	0.835	0.883	0.582	0.884
recall	0.735	0.783	0.895	0.840
F-score	0.782	0.830	0.705	0.860

Table S2. The performance of CLIIQ and ISP in the second experiment, where at most two isoforms of each gene were expressed.

	CLIIQ		ISP	
	1 sample	5 samples	1 sample	5 samples
precision	0.859	0.857	0.540	0.890
recall	0.808	0.879	0.927	0.879
F-score	0.833	0.868	0.682	0.884

The comparison of performance of CLIIQ and ISP is shown in Table S1 and S2. When comparing ISP with CLIIQ on a single sample in both experiments, CLIIQ shows a higher precision but a lower recall than ISP. Both algorithms are able to take the advantage of multiple samples, as indicated by the increased F-scores using 5 samples compared with using 1 sample. When 5 samples are used, ISP outperforms CLIIQ in both precision and recall. Moreover, the precision and F-score of ISP improve drastically with a slight drop in recall as the number of samples increases, indicating that ISP is able to take full advantage of multiple samples.

4 An example of ISP/Cuffmerge/Cufflinks predictions using ENCODE RNA-Seq samples

Figure 1 presents an example of transcriptome assembly results between exon 10 and exon 11 of gene CBP85, using 6 RNA-Seq samples from different ENCODE cell lines. Both ISP and Cuffmerge predicted transcripts

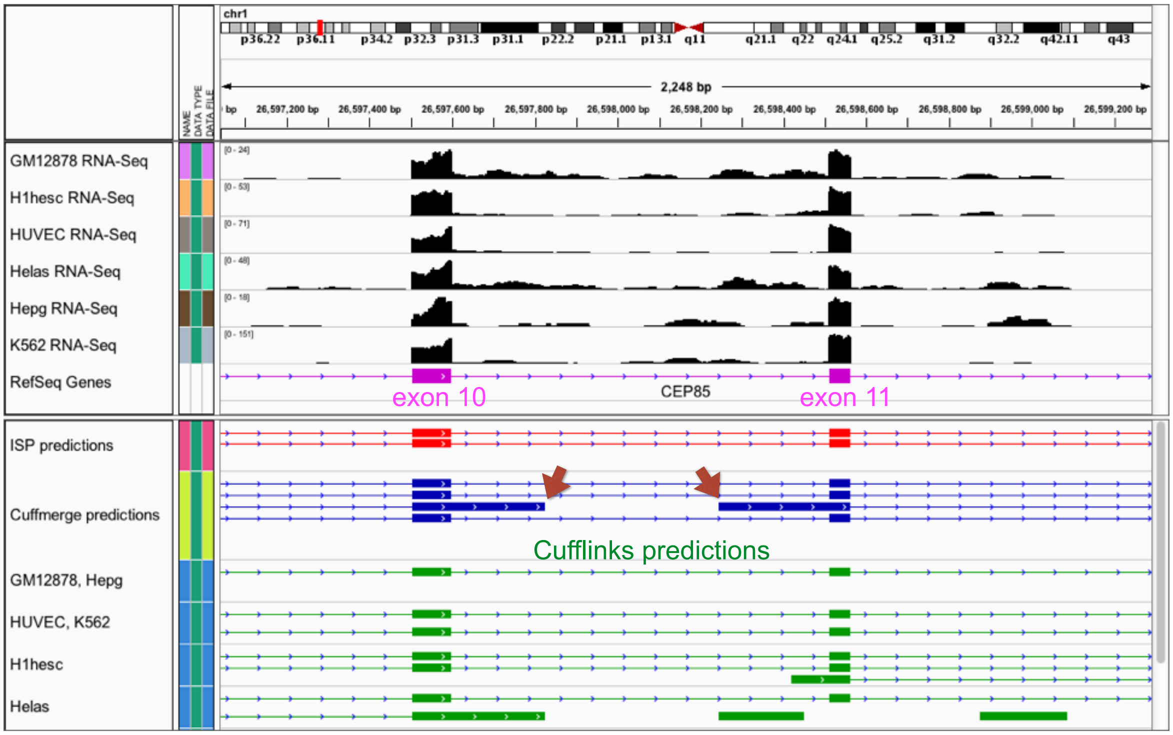

Fig. 1. Transcriptome assembly results of ISP, Cuffmerge and Cufflinks between exon 10 and exon 11 of gene CEP85. Tracks from top to bottom: RNA-Seq signals of 6 ENCODE cell lines (black), RefSeq genes of CEP85 (magenta), ISP predictions (red), Cuffmerge predictions (blue), and Cufflinks predictions on 6 individual samples (green). Two Cuffmerge predictions are probably false positives (indicated by the red arrows) and might have resulted from assembly errors of Cufflinks in individual samples.

that are consistent with the CBP85 RefSeq records. However, Cuffmerge predicted two additional transcripts, one ends with exon 10 and the other begins from exon 11. Both transcripts are probably false positives because they correspond to intron retention signals in some of the samples. A closer look at Cufflinks predictions on individual samples reveals the origins of two putative false positives. Exon 11 was predicted incorrectly in one transcript in H1hesc, while exon 10 was predicted incorrectly in on transcript in Helas. The strong intron intention signal in Helas also led to predict a few short single-exon isoforms, which might have also affected the results of Cuffmerge.

5 Sensitivity and precision of alternative splicing

We studied the sensitivity and precision of both ISP and Cuffmerge in detecting alternative splicing (AS) events in the presence of noisy reads. In this study, all AS sites from UCSC human known transcripts were compared with the AS sites that appear in predicted transcripts by ISP or Cuffmerge. We calculated both sensitivity and precision in the same way as in isoform inference presented in the Results section. More specifically, two AS sites are matched if their coordinates are identical. If K of M predicted AS sites match K of N known AS sites, then the sensitivity and precision are defined as K/N and K/M, respectively.

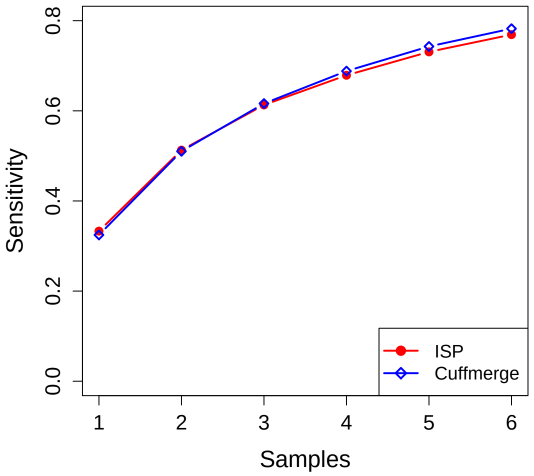

Fig. 2. The sensitivity in alternative splicing event detection.

Figure 2 and 3 show the sensitivity and precision of both ISP and Cuffmerge, by using different numbers of samples. Consistent with the trends in isoform inference (Figure 5), sensitivity increases and precision decreases slightly as more samples are used. Compared with Cuffmerge, ISP has a similar level of sensitivity but higher level of precision, suggesting that ISP is able to recover AS sites with a lower false positive rate, without sacrificing the sensitivity.

References

1. Lin, Y.-Y., Dao, P., Hach, F., Bakhshi, M., Mo, F., Lapuk, A., Collins, C., Sahinalp, S.C.: CLIIQ: Accurate Comparative Detection and Quantification of Expressed Isoforms in a Population Algorithms in Bioinformatics. Lecture Notes in Computer Science, vol. 7534, pp. 178–189. Springer, Berlin, Heidelberg (2012). Chap. 14

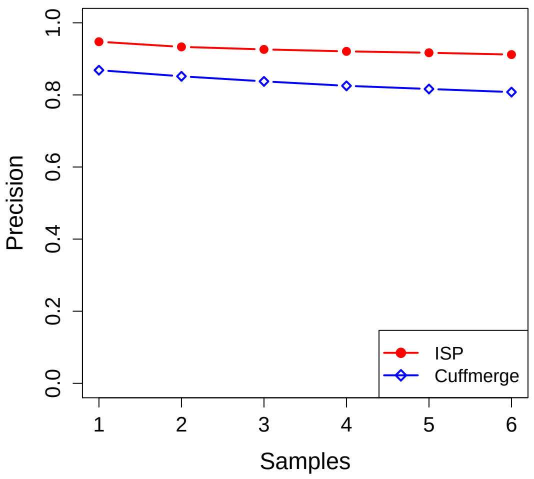

Fig. 3. The precision in alternative splicing event detection.
